# Young People’s Preferences for an Asthma Self-Management App Highlight Psychological Needs: A Participatory Study

**DOI:** 10.2196/jmir.6994

**Published:** 2017-04-11

**Authors:** Dorian Peters, Sharon Davis, Rafael Alejandro Calvo, Susan M Sawyer, Lorraine Smith, Juliet M Foster

**Affiliations:** ^1^ School of Electrical and Information Engineering Faculty of Engineering University of Sydney Sydney Australia; ^2^ Clinical Management Group Woolcock Institute of Medical Research University of Sydney Sydney Australia; ^3^ Royal Children’s Hospital Centre for Adolescent Health Melbourne Australia; ^4^ Department of Paediatrics The University of Melbourne Melbourne Australia; ^5^ Murdoch Childrens Research Institute Melbourne Australia; ^6^ Faculty of Pharmacy University of Sydney Sydney Australia

**Keywords:** asthma, mobile applications, quality of life, mental health, adolescents, chronic disease, mhealth, participatory design

## Abstract

**Background:**

Although the prevalence of mental illness among young people with asthma is known to be twice the rate of the wider population, none of the asthma apps reported have acknowledged or attempted to include psychological support features. This is perhaps because user involvement in the development of asthma apps has been scarce. User involvement, facilitated by participatory design methods, can begin to address these issues while contributing insights to our understanding of the psychological experience associated with asthma and how technology might improve quality of life.

**Objective:**

The goal of this participatory user research study was to explore the experience, needs, and ideas of young people with asthma while allowing them to define requirements for an asthma app that would be engaging and effective at improving their well-being.

**Methods:**

Young people aged 15-24 years with doctor-diagnosed asthma were invited to participate in a participatory workshop and to complete a workbook designed to elicit their thoughts and ideas about living with asthma, technology use, and the design of an app. Participants generated a number of artifacts (including collages, concept maps, and paper prototypes) designed to reify their ideas, tacit knowledge, and experience.

**Results:**

A total of 20 participants (mean age 17.8 years; 60%, 12/20 female) representing a range from inadequately to well-controlled asthma completed a workbook and 13 of these also took part in a workshop (four workshops were held in total), resulting in 102 participant-generated artifacts. Theoretical thematic analysis resulted in a set of personal needs, feature ideas, and app characteristics considered relevant by young people for an asthma support app. The data revealed that psychological factors such as anxiety, and impediments to autonomy, competence, and relatedness (as consistent with self-determination theory [SDT]), were considered major influences on quality of life by young people with asthma. Furthermore, the incorporation of features pertaining to psychological experience was particularly valued by participants.

**Conclusions:**

In addition to practical features for asthma management, an app for young people with asthma should include support for the mental health factors associated with lived experience (ie, anxiety, lack of autonomy, and social disconnectedness). We show how support for these factors can be translated into design features of an app for asthma. In addition to informing the development of asthma-support technologies for young people, these findings could have implications for technologies designed to support people with chronic illness more generally.

## Introduction

### Asthma Prevalence and Psychological Comorbidities

Asthma is considered the 14th most important disorder in the world in terms of the extent and duration of disability, affecting over 300 million people [1]. Furthermore, people with asthma are significantly more likely to experience mental illness, for example, being six times more likely to develop anxiety disorder [2].

Young people (aged 12-24 years) with asthma are also significantly more likely to suffer psychological dysfunction [3], including greater levels of depression, anxiety, risk-taking behavior, and social isolation as compared with their peers without asthma and also as compared with people with asthma of other age groups [1,3]. Adolescence is a complex period including issues of puberty, personal identity, sexuality, and vocational decision-making and having a chronic illness adds to the psychosocial burden of adolescence, for example, through the requirement to take on more responsibility for asthma self-management and dealing with gradual transition from pediatric to adult care [4].

On the basis of the severity of asthma and its psychological comorbidities, and in light of survey data showing a desire among young people for mobile-based asthma support, Blanchard et al have called on researchers to “develop tailored Web-based and mobile apps to support the wellbeing of young people with asthma, targeting asthma control, mental health, and general wellbeing side-by-side.” They also report that 83% of young people with asthma surveyed reported using a mobile phone “every day or almost every day.” Nevertheless, no asthma app in the existing research literature has attempted to address any of the psychological comorbidities associated with the illness [5-7].

### Participatory Design as a Method for User-Involvement

In this paper, we describe results from the participatory design phase of an app intended to improve the well-being of young people with asthma. We refer to young people aged between 10 and 24 years, which include adolescents aged between 10 and 19 years [8].

A participatory design approach was selected to overcome the typical lack of user-involvement reported in the development of existing health and asthma apps [9,10]. The need for end-user involvement from the outset is now acknowledged as critical to a successful health app development process [11-13]. 

Participatory design as a methodology, and general user-centered orientation, provides methods for the direct involvement of end-users in the codesign of technologies. It is distinct from other forms of user-centered design in that it positions designers as facilitators and views users as active cocreators of the solution [14]. Participatory design has been used as a method for involving patients or end-users in the design of health technologies and services in order to develop tools that are more likely to be engaging, relevant, and effective [15,16]. The approach has also become increasingly popular when involving adolescents in technology design [17-19].

### Self-Determination Theory as a Model for Well-Being and Technology Engagement

Self-determination theory (SDT) is a psychological model of motivation that has been successfully applied to understand and predict psychological well-being [20,21] specifically with respect to the satisfaction of three basic psychological needs: *autonomy* (to experience volition; act in accord with one’s interests and values), *competence* (to feel effective), and *relatedness* (to interact with, feel connected to, and care for others) [22]. Health intervention research utilizing SDT has shown that the more support for autonomy an individual has for their health, the more likely they are to achieve improved health outcomes, such as symptom control and quality of life [23]. The SDT model has also been shown to explain and predict engagement with technologies [24,25]. Given the usefulness of SDT to both supporting health outcomes and engagement in technology design, it serves an ideal foundation to a “positive computing” [26] approach (a topic of this special issue) to designing health apps that support well-being as well as health outcomes.

Ryan and Deci [27] also provide characteristics of environments (ie, designable features) that can be considered supportive or hindering to autonomy, competence, and relatedness (ie, goal choice, positive feedback, informational rewards), thus providing some initial practical guidance to designers. As such, SDT provides a uniquely useful framework for both the qualitative analysis of psychological experience and for translating insights from this analysis into actionable design specifications for technologies.

Autonomy is of special importance to the health community. It has not only been implicated as critical to mental health [27,28] but also is now considered critical to doctor-patient relationships and to health organizations. Taking autonomy-supportive approaches to patient interaction (eg, enabling patients to make informed decisions, active listening, taking a patient’s personal circumstances into account) is now considered a guiding principal of biomedical ethics [27,29]. Given its importance for doctor-patient relationships and to health organizations, it stands to reason that health *technologies* should also be designed in autonomy supportive ways. This paper contributes to a growing body of literature [30] on how human autonomy can be supported through technology design, in this case through the design of an app which aims to support the psychological well-being of young people with asthma.

In this paper, we aim to help address the gap in psychological support for asthma by (1) presenting qualitative results of the psychological experience of asthma as reported by young people, and (2) providing examples of how an app for asthma can be designed to support psychological factors.

## Methods

### Recruitment

Using convenience sampling, young people aged 15-24 years were invited to participate in the study via communications sent to social media, university websites, community pharmacies, sporting clubs, high school nurses, asthma educators, and organizations providing services for young people located in the metropolitan areas of Sydney and other parts of Australia. Eligibility criteria for the study included age (15-24 years), residency in Australia, and doctor-diagnosed asthma (self-reported). Level of asthma control was determined via an Asthma Control Questionnaire (ACQ) given after initial recruitment. Participants were offered a Aus $50 store voucher as compensation for their time. This research was approved by the Research Ethics Committee at the University of Sydney.

### Participatory Design Workshops and Workbooks

Activity-based workshops (2.5 h each) were carried out with young people with asthma. During each workshop, a user experience specialist-guided participants through a series of activities designed to elicit (1) emotions, perceptions, and needs related to being a young person with asthma and (2) design ideas and preferences for an app to support young people with asthma in a relevant and engaging way.

Activities were selected in order to fulfill a series of user research questions defined by the research team at the start of the project. Activities were drawn from the literature in participatory design [31,32] and included collaborative collage, individual concept mapping, group ideation, and paper prototyping. Data collected included participant-generated artifacts from each of the activities (ie, collages, concept maps, and screen designs), field notes, and audio recordings with transcriptions of each workshop.

Workbooks were also received from young people with asthma. The workbook was originally intended as a sensitization tool [14] to be completed by participants before the workshop. However, as the difficulty in recruiting participants to workshops became apparent, the workbook was also repurposed as an alternative method of participation for individuals who could not attend workshops and allowed the researchers to collect data from a broader sample by including those outside the state.

Workbook data included information on commonly used apps, and perceptions and experiences to do with health.

Issues to do with mental health and psychological factors were not prompted in either the workshops or workbooks but emerged independently.

### Questionnaires

Each participant completed a questionnaire that included questions on demographic characteristics and the 6-item Asthma Control Questionnaire (ACQ), a standard validated scale to measure the range of asthma control among participants (scored 0 [best] and 6 [worst]; ≤0.75: well-controlled asthma; ≥1.50: inadequately controlled asthma [33].

### User Feedback on a Prototype

Consistent with participatory design methods, user evaluation was conducted with nine of the original workshop participants on a high-fidelity prototype after the initial design phase. These user testing sessions were audiorecorded for later analysis.

The app’s content was also reviewed for accuracy of medical content by a team of clinicians.

### Crowdsourcing

In order to allow young people with asthma to name the app, an invitation was posted to the Young People with Asthma Facebook community inviting submissions for an asthma app name. The research team then selected from the options contributed (based on distinctiveness) and tested acceptability of the name during the user testing phase.

### Data Analysis

Using theoretical thematic analysis [34] and consistent with methods for the analysis of generative participatory data [14], the text and imagery from the artifacts and workbooks were analyzed by a member of the research team and categorized into themes as detailed below.

Although the user research was exploratory and open in nature, it was conducted toward a specific outcome (the development of an effective app) and therefore analysis was guided by the research questions in support of that outcome. The theoretical thematic analysis focused on those categories of data that would inform development, namely: (1) categories of features and characteristics of an ideal app, and (2) categories of lived experience that could be addressed via technology. For the former, categories were straightforward feature types, for example, a category “social features” included “chat,” “forum,” and “story sharing,” whereas a category “Feedback” included “encouraging messages,” “evidence of achievement,” and “evidence of progress.”

For the more diversely articulated and abstract descriptors of lived experience, data was categorized using SDT as a framework in order to link elements of experience as reported by participants to evidence-based well-being constructs, thus facilitating the translation of findings to relevant interaction designs (as mentioned earlier, SDT literature provides some links from well-being constructs to technology design features). A similar application of SDT to content analysis has been used in the context of smoking [35]. For the collage activity involving imagery, participants were asked to describe the images they chose and these descriptive words were included in analysis.

## Results

### Demographics

A total of 48 young people contacted the research team expressing an interest in participation; of which 28 chose not to participate due to various factors such as geographical location, school, or work schedules and 20 young people (mean age 17.8 years [range 15-24]; 12 female) with a range of asthma control (ACQ: 1.5 [0.2-4.5] and 12 prescribed a preventer inhaler) completed a workbook and 13 of these also took part in a workshop (workshops consisted of between 2 and 7 participants) between May 2015 and November 2015. Of the 20, 7 only completed a workbook, whereas 13 completed both a workshop and workbook. More demographic detail is given in Table 1.

**Table 1:** Participant demographics.

Participants		N=20
Age, years, mean (range)		17.8 (15-24)
Female, n (%)		12 (60)
**Occupational status, n (%)**			
	High school student	12 (60)
	University student	6 (30)
	Working	1 (5)
Socioeconomic indexes (median; range)^a^		5; 1-5
Asthma Control Questionnaire, mean (range)^b^		1.5 (0.2-4.5)

^a^Social disadvantage at home address: “Disadvantaged” SEIFA (Socioeconomic Indexes for Areas) Quintile ≤3, “Advantaged” SEIFA Quintile: 4-5 [36].

^b^Scored 0 (best) and 6 (worst); ≤0.75: well-controlled asthma; ≥1.50: inadequately controlled asthma [33].

A total of 12 submissions were received for the crowdsourced app naming campaign. The name “Kiss My Asthma” was selected from among the 12 by the research team, in consultation with Asthma Australia (national asthma charity and research funders) and based on criteria, such as distinctiveness from other app names and appropriateness to the goals of the app.

Qualitative analysis of 102 participant-generated artifacts (ie, collages, concept maps, and screen designs) yielded a set of user needs, goals, obstacles, and supports with respect to the lived experience of being young with asthma, as well as a list of preferred features and characteristics for an asthma app from the perspective of young people. Results showed clear evidence of a need to support psychological well-being for those with asthma, along with some specific ideas for how to do so within the context of an app, as detailed below.

### The Experience of Being Young With Asthma

**Figure 1:**
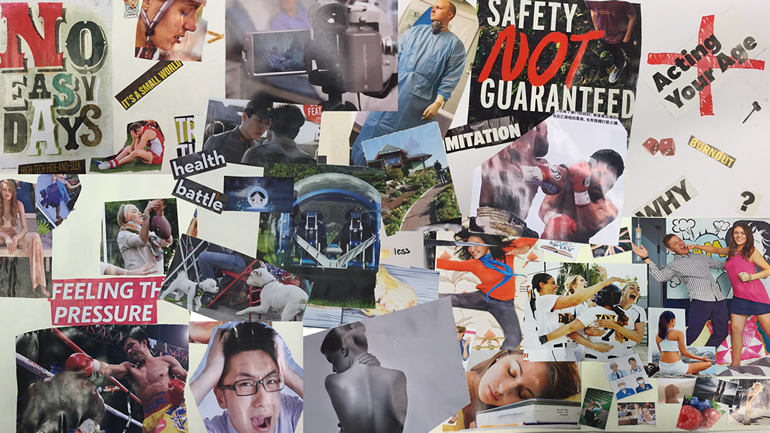
Combined imagery from workshop participant collages.

Workshop participants collaboratively created collages to represent “what it’s like to be a young person with asthma.” Figure 1 shows combined imagery from workshop participant collages and includes images depicting asthma as a “battle;” asthma as a source of stress, anxiety, and social exclusion; the need to put on a healthy façade; and asthma as a barrier to living like a “normal teenager.” The resulting collages and discussion overwhelmingly focused on the psychological experience of living with asthma (something unprompted and unanticipated by the researchers) and revealed key themes consistent with the basic psychological needs described by SDT. Examples of collage imagery depicting emotional experience as well as illustrative quotations are included below.

#### Autonomy: Lack of Control, Restrictions, and Uncertainty

Participants expressed feelings of lack of autonomy in the form of frustration and anxiety over lack of control, the inability to take part in activities, and uncertainty...

You never know what will happen, cause you never know when you’ll have an asthma attack.

female, 18 years

When it does happen, we become subject to the asthma attack and can’t really control our actions or defend ourselves.

male, 16 years

If you forget your (rescue) meds, you’re panicking the whole time.

female, 18 years

You’re in a bit of a bubble with what you can do, eat and touch.

female, 19 years

We want to be a bit more Zen, a bit more carefree (because having asthma is) pretty scary

female, 22 years

#### Competence: Not Keeping Up, Struggling, Feeling Unprepared

Participants also expressed feelings of lack of competence with respect to physical competence, keeping up with peers, managing their health, and feeling unprepared for asthma emergencies...

We still do it, it’s just a bit harder, it’s not as easy to keep up.

female, 18 years

l always struggled to keep up with others in training.

female, 18 years

Asthma’s holding us back.

male, 15 years

Asthma is the guy that’s punching you.

male, 17 years

It’s like you against asthma and it’s always there. I have the words “health battle” because I feel that really captures it.

female, 19

#### Relatedness: Being Left Out, Social Pretense, Isolation

Participants expressed lack of relatedness as feelings of isolation, distress at being left out, having to put on a healthy façade, and being different to peers.

You can’t act your age because of asthma.

female, 18 years

It’s not as easy to keep up(so you) feel left out.

female, 18 years

How we feel trying to navigate around growing up, cause it’s different—even going to school each day—you have to worry about a lot more things than other kids, even through the day.[female, 22 years]

You pretend you’re fine even when you’re not.

female, 18 years

Two different perspectives on people with asthma: the facade that we put on saying we’re healthy and fine, and then the more chaotic side.

male, 16 years

### Life Goals, Obstacles, and Supports

The participants reported a number of life goals via the individual concept-mapping activity. This contributed insights into the intrinsic motivations young people have to manage their asthma. Participants mostly reported shorter-term goals with some notable big picture aspirations. The majority of goals could be clustered into one of four themes: (1) study, (2) job and career, (3) health, fitness, and sport, and (4) spirituality, happiness, and meaning.

Common obstacles to goal achievement reported included asthma and related illness (ie, allergies and anaphylaxis that are common comorbidities in people with asthma), lack of money, and lack of motivation. Common supports reported included family, friends, and personal traits such as determination (previous research has shown the importance of support to successful asthma management [37]). A list of these themes, along with illustrative quotations and how participants related goal obstacles to asthma (when they did), are included in Table 2. Tables 3 and 4 show the obstacles and supports participants reported, respectively.

### Preferred Features

**Figure 2:**
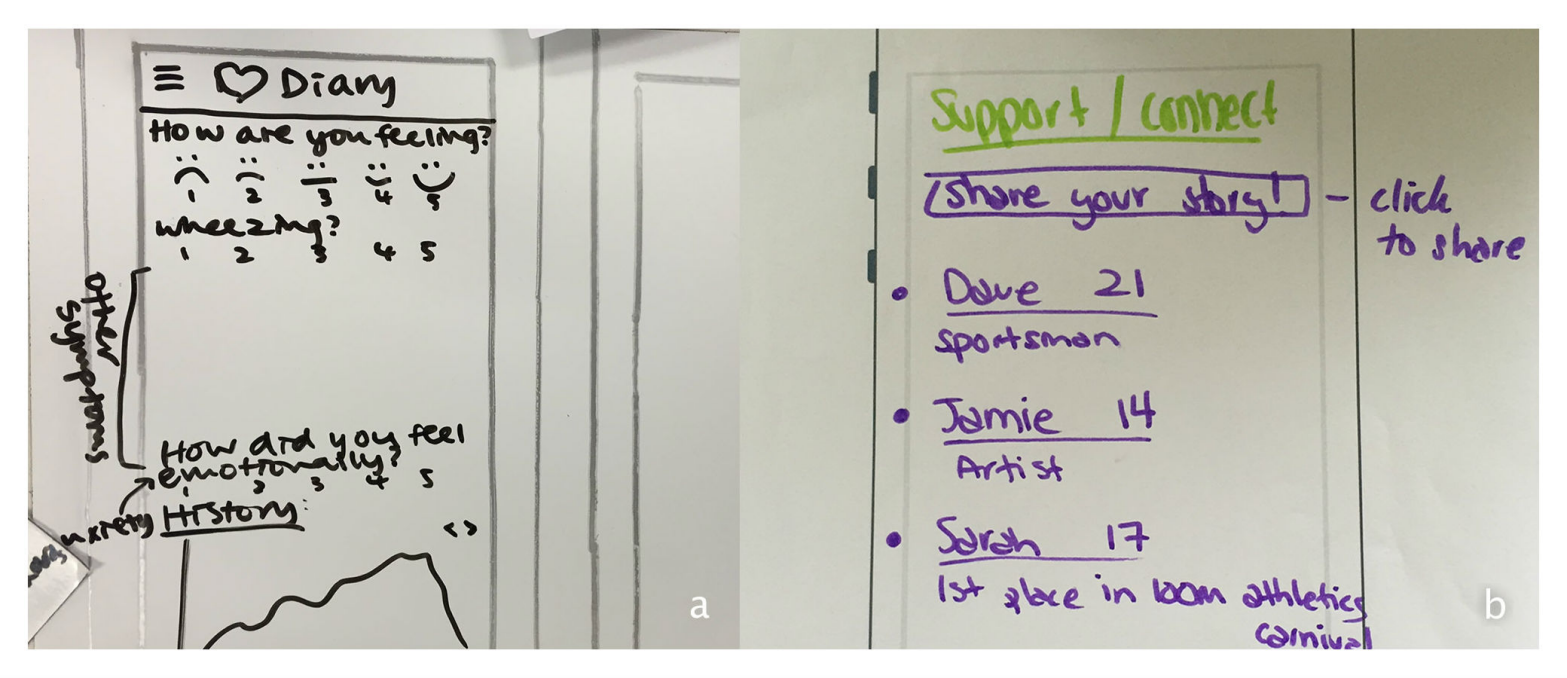
Example screen designs showing: a) mood tracking b) personal story sharing.

As part of a brainstorming activity, participants generated ideas for features that they believed would make the app practical, useful, and engaging. These are presented in Table 5 in a categorized list beside the psychological need (SDT construct) most directly supported by each feature. *Autonomy* is indicated for features that provide the user with choices, or access to tools providing a greater sense of control. *Competence* was selected for features that provide users with knowledge or skills that increase their capacity to handle situations. *Relatedness* was selected where features connect the user to others or contribute to a sense of belonging. Of course, many features contribute to more than one psychological need. The list is not intended to be definitive, but rather to help make explicit how practical design features can contribute to psychological experience in health apps. Figure 2 shows example screen prototypes created by participants.

**Table 2:** Goals in life.

Goal theme	Examples of goals as written by participants^a^	Relationship of goal to asthma
Study	“Finish HSC^b^,” “School leadership,” “Be a good student,” “Get a defense scholarship”	Asthma sabotaged exams
		Missed class when in hospital for asthma
		Couldn’t pass physical exam for defense scholarship
Job and career	“Become a professional footballer,” “Become a manager at Maccas [sic],” “Become a doctor (ophthalmic surgeon),” “Get a job that I enjoy and love,” “Become a child carer,” “Be an archaeologist”	Career goal requires travel to countries with different asthma-inducing climates
		Exceptional fitness required to be a professional athlete
Health, fitness, and sport	“Be physically fit—able to play sport for a longer time without getting tired,” “STAY ALIVE,” “Get new hearing aids,” “Get a good night’s sleep,” “National champion fencing and Olympics,” “Be a good netball player”	Asthma limits athletic performance
		Asthma limits ability to maintain physical fitness
		Asthma interrupts sleep
Spirituality, happiness, and meaning	“Achieve happiness,” “Live a long and happy life,” “Becoming more like Jesus,” “Caring for others in a genuine loving way,” “Charity work”	No direct connections to asthma were reported for these goals

^a^All text quoted verbatim from the contributions made by participants during concept mapping.

^b^HSC: Higher School Certificate.

**Table 3:** Obstacles in life.

Obstacle theme	Examples of obstacles as written by participants^a^
Asthma and health	“Asthma,” “Exhaustion,” “Bringing my Ventolin,” “Emergency situations where I need to run,” “Allergies”
Money and circumstances	“Money (lack thereof),” “Time (lack of),” “Constraints: (time-based, family-based, etc.)”
Lack of motivation	“Laziness,” “Lack of motivation,” “Not caring about certain [school] subjects”
Study and work	“Academic marks,” “Experiments not working,” “Inexperience”
Personal faults	“Not managing my time well,” “Selfishness, envy”

^a^All text quoted verbatim from the contributions made by participants during concept mapping.

**Table 4:** Supports in life.

Support theme	Examples of supports as written by participants^a^
Other people	“Family or friends,” “Netball team,” “Coaches”
Medicine or health	“Medication,” “Puffer,” “Training my body to be fitter”
Circumstances	“My school,” “Australia,” “Opportunities”
Personal effort	“Working hard,” “Studying,” “Getting involved [in extra-curricular activities],” “Reminding myself to be proactive”
Personal traits	“Personal nature as a caring person,” “determination,” “perseverance”
Faith or spirituality	“Jesus”

^a^All text quoted verbatim from the contributions made by participants during concept mapping.

Although the features suggested by participants may address psychological needs, it is interesting to note that, with the exception of mood tracking, participants did not explicitly connect the psychological factors they expressed so strongly during the collage activity, to app feature ideas. This is probably because there is little precedent for providing psychological support among health apps, or apps for young people generally. However, when the development team added an explicit mental health feature (a “managing anxiety” section), participants during user testing expressed strong approval, requesting the section be expanded (detailed below).

**Table 5:** List of suggested features linked to psychological needs.

Feature		Psychological need supported
**Profile**		
	Medical history and treatment summary	Competence
	Medication list	Competence
	Customizable to the particular health characteristics or health needs of individual users	Competence
**Reminders**		
	Medication alerts (eg, “It’s time for 2 puffs of preventer”)	Competence
	Feature that allows users to find their inhaler “Find my puffer”	Autonomy or Competence
	Location-based reminders (“alert! You are outside of your home. Do you have your puffer?”)	Autonomy or Competence
**Tracking**		
	Symptom diary (wheezing scale, coughing, and so on)	Competence
	Causes or triggers diary	Competence
	Asthma attack diary (“log an attack”)	Competence
	Mood diary	Competence
	Health over time overview graph	Competence
	Step count	Competence
	Overall log feedback: “Your attacks have been more frequent lately, maybe you should check with your doctor”	Competence
	Log reports to show your doctor	Autonomy or Competence
**Social**		
	In-app chat with other people with asthma	Relatedness
	Q&A forum	Competence
	Share personal experiences with others	Relatedness
	Share with friends (connect with friends to share achievements and encourage each other)	Relatedness
	Share your story (inspiring stories from young people overcoming an asthma challenge)	Relatedness
**Emergency support**		
	GPS^a^ tool to find the nearest emergency service	Autonomy or Competence
	“How to handle an asthma attack” screen to show others	Autonomy
	Asthma attack management sequence for user	Autonomy or Competence
	Emergency contacts	Autonomy or Competence
	Off-line mode or not Internet-dependent (especially for emergency info)	Autonomy
	Emergency info available from lock screen	Autonomy
**Expert access and information**		
	Chat with a friendly professional	Competence
	Automated chat (for off-hours)	Competence
	Expert and how-to videos	Competence
	List of specialists near you	Autonomy
	Personalized asthma facts and statistics (based on your profile)	Competence
	Information on how to prevent an attack	Competence
**Feedback and motivational features**		
	Support messages, badges, points for good behavior (“you’ve been doing well—go reward yourself!”)	Competence
	Daily motivations (quotes)	Competence
	Competing with friends or anonymous strangers	Relatedness

^a^GPS: Global Positioning System.

### User Feedback on a Prototype: “More Support for Mental Health”

User testing of an app prototype based on the data gathered at the user workshops occurred 8 months after the initial codesign phase (the gap between phases was a result of the challenges of recruiting minors including ethics committee approval, parental consent, and logistics), with nine of the original workshop participants. Feedback from the user testing was highly positive with all participants reporting that they would recommend the app to a friend. Moreover, a number of testers independently expressed approval of the mental health support features in the app, in particular, the information section on managing anxiety, and went further to suggest the section be expanded with practical exercises and tools for self-management. For example, one female participant (aged 22 years) stated during an individual user test...

I think anxiety is quite a big thing when you have asthma...There’s not a lot of information out there about how to deal with it. As a child, when I had bad asthma I had to attend the hyperventilation clinic to teach me how to manage anxiety...So if there was a bit more information about it...even basic exercises that you could try, to keep your asthma, or even your anxiety, under control.

When you’re dealing with asthma and then it’s like “go speak with your doctor;” “go do this and that.” But if it’s small things you could learn to do yourself I think that probably is a good thing. Because anxiety is not a thing we spoke about with asthma especially when I was young. It’s becoming more understood now, but it’s actually quite a big problem. Anxiety in general is a big thing that we don’t often look at.

A second female participant (aged 19 years) provided similar advice:

Perhaps, on the managing anxiety thing, add other options. For example, something you enjoy, have a bath or listen to some music, do a craft. Something other than “talk to your doctor,” etc. All of those are great, but not when you’re having an asthma attack.

Finally, one male participant’s (aged 16 years) reference to stress provides evidence for how even esthetic design decisions can link to well-being factors:

Stress has been proven to exacerbate asthma and this relaxes me with its colorsthe blue and the cloud.

## Discussion

### Principal Findings

This participatory study revealed that the lived experience of young people with asthma includes a number of psychological factors that can—–and should, according to participants—be supported via an asthma app.

Although the participatory approach posed challenges from a recruitment perspective, its benefits became clear when factors relating to psychological experience emerged. It was through generative participatory practices (in particular, collaborative collage and concept mapping) that difficult-to-articulate matters of psychological experience came to light. In other words, had user involvement not been facilitated through a participatory approach, we arguably would not have come to understand the importance to young asthma sufferers of psychological support needs, and their desire for these to be addressed via technology.

Indeed, the name “Kiss My Asthma,” which was elicted from young people with asthma, reflects a highly autonomous, if irreverent, attitude toward asthma as an adversary (a notion expressed by participants in workshops, eg, “Asthma is the guy that’s punching you in the face”). It also demonstrates the importance of user input when developing autonomy supportive apps since it seems unlikely that any researcher or clinician would have named the app in such a way.

The interest in psychological support features, including anxiety management and mood tracking, may result from the lack of attention given to mental health comorbidities in adolescents with asthma. Although it may be especially pertinent for adolescents with asthma due to the anxiety that breathing difficulties can engender, the desire for technology-based mental health support may also have important implications for adolescents with other chronic illnesses and requires further research.

The high user satisfaction feedback received during prototype testing provides early evidence for the success of the participatory methods, and support for the consideration of psychological factors in the app.

### Translating Findings on Psychological Factors to Design Features

The psychological factors revealed during participatory design workshops required further analysis to translate into actionable design specifications. The literature in positive computing provided an approach to designing technology to support psychological well-being that includes structuring ideation around individual well-being determinants [26]. Mental health factors reported in this study (eg, anxiety, panic, social disconnectedness) were clustered as impairments to autonomy, competence, and relatedness as consistent with SDT. SDT was useful both for categorizing user needs, as well as for linking these needs to evidence-based well-being determinants that could become the focus of design.

**Figure 3:**
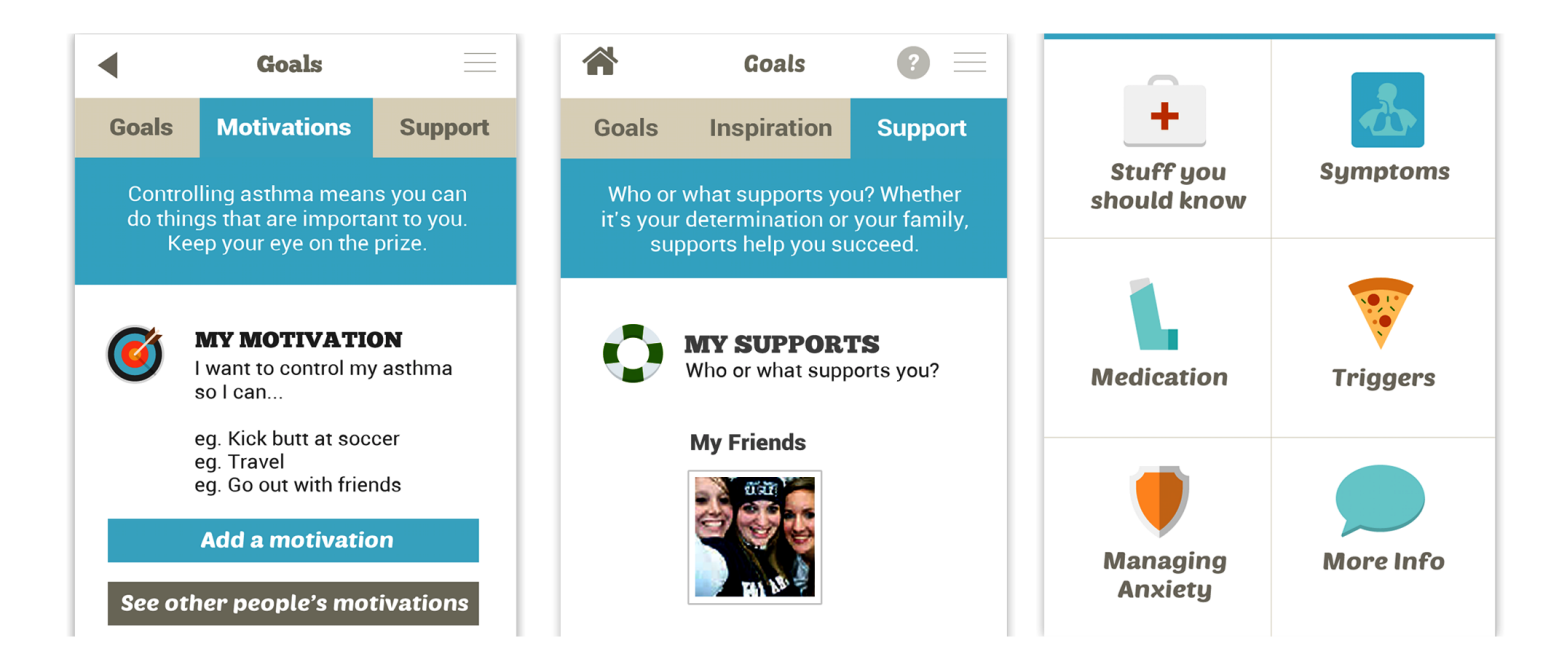
Example app screens: (left) “My Motivations,” (middle) “My Supports,” and (right) “Managing Anxiety” in “About Asthma”.

The research team, which includes two psychologists and an interaction and user interface designer, further developed the psychological support features for the needs for autonomy, competence, and relatedness expressed by the young people (Table 6). This example not only shows how the use of a motivational model and user-experience methods can work in synergy to guide design for health technologies, but also functions as an example of positive computing in which ideation is based on well-being determinants (in this case those identified by SDT). Figure 3 shows three example screens from the prototype app, which relate to these supportive design features.

**Table 6:** Psychological support features.

Psychological factor	Supportive design features
Relatedness (Make salient user belongingness to a larger community while maintaining privacy and safety)	Convey the message “you are not alone” by including text on the prevalence of mental health issues among young people with asthma.
	Provide users with a place to upload images of those who support them.
	Allow optional anonymous sharing of user motivations for asthma management. Allow users to browse a gallery of other users’ shared motivations (*these are currently moderated for safety*).
	Provide the app dialogs as if they were coming from a supportive friend (delivered by characters rather than by “the phone”).
Autonomy and competence (Provide tools that will allow users to feel more in control and better able to handle asthma impacts and emergencies; support autonomous motivation, based on personal values and goals [38])	Provide strategies and tips for better asthma control.
	Scaffold goal-setting to support agency and improve asthma control.
	Scaffold the identification of autonomous motivations for managing asthma (by allowing users to record and share their own autonomous motivations and to view others).
	Provide an information section on managing anxiety and panic attacks.
General support for mental health	Provide a calming esthetic (ie, light color palette, cloud metaphor, “Cute monster” characters).
	Incorporate subtle humor as part of dialogs to lighten the otherwise stressful context of asthma control.
	Provide links to mental health support lines and resources for young people.

The impact of practical features (eg, symptom tracking) on a sense of autonomy is further supported by recent research into using asthma app features to promote adolescent self-management through autonomy [39].

### Limitations and Future Work

The participant cohort, while representative with respect to age, gender, and asthma control, was limited with respect to social disadvantage (most participants lived in a socially advantaged location and were urban or suburban students of either a university or private high school). Further studies targeting other demographics would contribute to generalizability. Our study volunteers may have been more engaged with their disease than the wider population; their preferences may not fully represent individuals less engaged with asthma.

A number of constraints intrinsic to app development projects, including limited access to developers and a limited time frame, meant that the data could only be analyzed by one researcher, and then reviewed by a team of researchers, before being implemented for development. Although this approach allowed research insights to inform design and development, additional analysts would provide greater reliability to the findings. Some confirmation of reliability was provided by the high satisfaction rates reported during prototype testing, but owing to the small sample size, further evaluation would strengthen these findings as well.

In a next phase of the project, we will evaluate the impact of app features on quality of life (and against measures of autonomy, competence, and relatedness) as well as on asthma control outcomes as part of a clinical trial. Contingent on positive outcomes from the clinical trial, we will release the app to the public on World Asthma Day, May 2, 2017. It is hoped that following public release, having an access to usage data from a significantly larger audience for the app will allow us to conduct a larger scale quantitative study on the use and impact of various features, including those that support intrinsic motivation, mental health, and well-being.

### Comparison With Prior Work

Cushing et al [40], Roberts et al [41], and Burbank et al [42], recently reported on the use of mHealth for asthma support but each with specific foci, namely, a sensor device for adherence-support, communication with friends and caregivers, and asthma action plans, respectively. None of these employed participatory codesign methods. Furthermore, there are no reports of asthma apps that have taken into consideration the psychological factors associated with the disease.

### Conclusions

In this study, participatory design methods were used to (1) elicit descriptions of the lived experience from young people with asthma, (2) identify psychological support features and characteristics for an app for asthma from the perspective of young people, and (3) provide young people with the opportunity to guide and cocreate an app to support their asthma self-management and well-being. The resulting prototype was highly rated during user feedback.

In addition to informing the development of asthma-support technologies, this research provides a practical example of how health technologies in general can be designed to support autonomy, competence, and relatedness. Moreover, our finding that there is a desire for technology-based mental health support among young people with asthma could have implications for adolescents with other chronic illnesses.

## Abbreviations

ACQ: Asthma Control Questionnaire
SDT: self-determination theory
